# Association between skeletal muscle mass and mammographic breast density

**DOI:** 10.1038/s41598-021-96390-9

**Published:** 2021-08-18

**Authors:** Kwan Ho Lee, Seoung Wan Chae, Ji Sup Yun, Yong Lai Park, Chan Heun Park

**Affiliations:** 1 Department of Surgery, Bucheon St. Mary’s Hospital, College of Medicine, The Catholic University of Korea, Seoul, Korea; 2 Department of Pathology, Kangbuk Samsung Hospital, Sungkyunkwan University School of Medicine, Seoul, Korea; 3 Department of Surgery, Kangbuk Samsung Hospital, Sungkyunkwan University School of Medicine, 29 Saemunan-ro, Jongno-gu, Seoul, 03181 Korea

**Keywords:** Breast cancer, Risk factors

## Abstract

Mammographic density (MD) of the breast and body mass index (BMI) are inversely associated with each other, but have inconsistent associations with respect to the risk of breast cancer. Skeletal muscle mass index (SMI) has been considered to reflect a relatively accurate fat and muscle percentage in the body. So, we evaluated the relation between SMI and MD. A cross-sectional study was performed in 143,456 women who underwent comprehensive examinations from 2012 to 2016. BMI was adjusted to analyze whether SMI is an independent factor predicting dense breast. After adjustment for confounding factors including BMI, the odds ratios for MD for the dense breasts was between the highest and lowest quartiles of SMI at 2.65 for premenopausal women and at 2.39 for postmenopausal women. SMI was a significant predictor for MD, which could be due to the similar growth mechanism of the skeletal muscle and breast parenchymal tissue. Further studies are needed to understand the causal link between muscularity, MD and breast cancer risk.

## Introduction

Mammographic density (MD) reflects the relative amount of connective and epithelial tissue (dense area) and fat tissue (non-dense area) in the breast^1,2^. It is known that the risk of breast cancer varies four to six times, based on the MD^1^. The difference in MD may reflect the effects of hormones and growth factors and might be related to the causal pathway of breast cancer^3^. Additionally, because cells at risk of turning malignant are mainly located in the fibro glandular tissue, the total amount of this tissue can also be considered as a risk factor for breast cancer^4^. Therefore, MD is a significant predictor for the risk of breast cancer, and factors influencing breast density might also contribute to the risk of breast cancer. However, the biological basis for determining MD is still unclear^3^.

There have been many studies on the factors related to breast density; however, different results have been reported due to heterogeneity and the small number of subjects examined and different statistical methods used for the analysis^5–14^. Increasing age and post-menopausal state with decreased estrogen levels is known to be a major determinant of MD^15^. Anthropometric factors, such as body mass index (BMI), waist–hip ratio, and waist circumference are also known to be related to MD^5–9^. In epidemiological studies, the most widely used index to evaluate body fat is BMI, which is calculated as the weight (kg) divided by height squared (m^2^). However, considering the inverse association between BMI and MD and the positive association between MD and breast cancer risk, the positive association between BMI and breast cancer risk remains unexplained in postmenopausal women^16,17^. The association between obesity and breast cancer risk in premenopausal women is not consistent across studies^18,19^. We supposed that the inconsistency could be because anthropometric factors used in previous studies are indicators of weight or mass including both muscle and fat, and these parameters do not reflect the respective amounts of each of muscle and adipose tissue.

We obtained the skeletal muscle mass index (SMI) using an equipment that measures the ratio of adipose tissue to muscle and evaluated the association between SMI and MD. Moreover, SMI indicates not only the proportion of skeletal muscle in the body, but are also considered better markers for assessing adiposity and its metabolic consequences compared to BMI. This is because SMI reflects a relatively accurate fat percentage in the body. Therefore, we analyzed whether the SMI can be a better predictor for MD, compared to the other anthropometric parameters used in previous studies. In addition, by attempting to adjust BMI in the analysis of the relationship between SMI and MD, it was intended to identify the cause of the paradoxical relationship between BMI and MD.

## Methods

### Study population

This study was a retrospective analysis of the data of 291,636 patients who underwent comprehensive annual or biennial examinations from 2012 to 2016 at the Samsung Healthcare Center in Seoul and Suwon, South Korea. Mammography was included as a part of the basic examination for all women over 30 years of age. The participants were employees of various companies, and were required to undergo an annual or biennial mandatory screening, which is required by the Industrial Safety and Health Law in Korea. The spouses of some male employees were also included due to the company’s welfare policies, and some women were self-registered. Of the data of 291,636 patients, if any participant underwent multiple examinations through several years (*n* = 145,898), the data at first year was used for analysis. We also excluded participants with missing anthropometric measures (*n* = 1387), and those with incomplete mammography (*n* = 895). Finally, a total of 143,456 women were included in the study. This study was approved by the Institutional Review Board of Kangbuk Samsung Hospital (KBSMC 2018-02-056), which waived the requirement for informed consent due to the use of deidentified data obtained as part of routine health screening exams, retrospectively. All methods were performed in accordance with relevant guidelines and regulations.

### Measurement of breast density by mammography

Mammograms were performed using a full-field digital mammography system (Senograph 2000D/DMR/DS, General Electric Company, Milwaukee, WI, USA). MD was assessed from the craniocaudal and mediolateral oblique views of the each breast, using the breast imaging reporting and data system (BI-RADS) with 4 categories: almost entirely fat, scattered fibroglandular densities, heterogeneously dense, or extremely dense by several experienced radiologists^20^. We defined dense breast tissue as extremely dense, while non-dense breast as almost entirely fat, scattered fibroglandular densities, heterogeneously dense for dichotomous analysis.

### Anthropometric measurements

Anthropometric measurements were obtained by the trained staff at the time of mammography. Height was measured to the nearest 0.1 cm using a stadiometer while the participant stood with heels together, arms to the side, legs straight, shoulders relaxed, and the head positioned so the gaze was straight ahead. Body weight was measured to the nearest 0.1 kg using a digital scale, with women in light clothing without shoes. Waist circumference (cm) was measured using a soft tape midway between the lowest rib margin and the iliac crest in a standing position. BMI was calculated as weight in kilograms divided by height in meters square.

The percentage of body skeletal muscle was estimated using a multifrequency bioelectrical impedance analyzer (BIA) with eight-point tactile electrodes (InBody 720, Biospace Co., Seoul, Korea). SMI was calculated as SMI (%) = skeletal muscle mass (kg)/body weight (kg) × 100, based on the methods used by Janssen et al.^21^.

### Other measurements

Data about the history and health-related behaviors, family history, menstrual and reproductive history were collected through a self-administered questionnaire. Blood samples were taken from the antecubital vein by trained staff after the participant had fasted for at least 8 h, to measure the biochemical parameters. Calcium level adjusted to albumin was calculated as serum calcium + 0.8 × (4 − serum albumin).

### Statistical analysis

Statistical analyses were performed using the STATA version 13.0 (StataCorp LP, College Station, TX, USA) and R version 4.0.3 with packages for relative weights (RW) analysis and scatter^22–24^. Chi-square test for categorical data and t test for continuous variables were used to compare the baseline characteristics between the groups. The associations of variables with breast density were evaluated using RW, which is a way quantify the relative importance of correlated variables in regression analysis and it could produce clear results even if the predictors have very high collinearity^23,25^. Pearson correlations and scatter plots were used for understanding the relationship between anthropometric factors, age and SMI^24^. Logistic regression analyses were conducted to investigate the impact on MD of anthropometric factors by estimating odds ratios (ORs) with 95% confidence internals (CIs). We adjusted for parameters like age, parity, age at menarche, history of estrogen replacement therapy, and calcium level adjusted to albumin. In addition, we adjusted for BMI to evaluate whether SMI was an independent factor for a dense breast. All reported *p* values are two tailed, and comparisons where *p* < 0.05 were considered statistically significant.

## Results

### Patient characteristics

Among the 143,456 participants, there were 115,013 premenopausal women (80.2%) and 28,443 postmenopausal women (19.8%). Table 1 shows the baseline characteristics of the study participants. The prevalence of dense breasts in premenopausal women was higher than that in the postmenopausal women. Dense breasts were positively associated with height, SMI, and estradiol level, and negatively associated with age, weight, BMI, waist circumference, vitamin D level, CA15-3, FSH, parity, and history of breast cancer in the premenopausal women. In postmenopausal women, dense breasts were positively associated with height, amount of alcohol consumption, SMI, estradiol level, and history of estrogen replacement therapy, while it was negatively associated with age, weight, BMI, age at menarche, waist circumference, vitamin D level, FSH, and parity. Regardless of the menopausal status, the factors that had large proportions in dense breast were similar.

**Table 1:** Characteristics and anthropometric measurements of participants by menopausal status and mammographic density.

Variables	All subjects (*n* = 143,456)	Premenopausal women			Postmenopausal women		
		Non-dense breast (*n* = 60,180)	Dense breast (*n* = 54,833)	*p* value	Non-dense breast (*n* = 25,570)	Dense breast (*n* = 2873)	*p* value
Age (years)	143,456	39.3 ± 5.7	37.3 ± 5.2	< 0.001	58.0 ± 7.5	50.7 ± 8.2	< 0.001
Height (cm)	143,456	160.7 ± 5.1	161.0 ± 5.0	< 0.001	156.2 ± 5.3	158.0 ± 5.2	< 0.001
Weight (kg)	143,456	58.4 ± 8.8	53.9 ± 6.9	< 0.001	57.9 ± 8.0	54.4 ± 7.6	< 0.001
Body mass index (kg/m^2^)	143,456	22.6 ± 3.2	20.8 ± 2.5	< 0.001	23.7 ± 3.1	21.8 ± 2.9	< 0.001
Age at menarche (years)	139,545	13.8 ± 1.5	13.8 ± 1.5	< 0.001	15.4 ± 1.8	14.8 ± 1.7	< 0.001
Amount of alcohol consumption (g/week)	124,558	5.6 ± 11.5	5.6 ± 12.1	0.566	3.9 ± 10.5	4.9 ± 10.3	< 0.001
Waist circumference	143,456	77.7 ± 8.2	73.0 ± 7.0	< 0.001	81.5 ± 8.4	75.6 ± 8.2	< 0.001
Skeletal mass index	143,456	37.2 ± 3.1	39.2 ± 3.0	< 0.001	35.7 ± 3.2	38.1 ± 3.3	< 0.001
Albumin	143,456	4.5 ± 0.2	4.5 ± 0.2	< 0.001	4.5 ± 0.3	4.5 ± 0.3	0.001
Vitamin D	97,072	14.7 ± 6.7	14.6 ± 6.7	0.043	17.9 ± 9.9	17.1 ± 8.8	0.001
CA 15-3	10,992	9.5 ± 4.4	9.2 ± 4.1	0.030	10.1 ± 4.9	9.8 ± 4.3	0.1944
Calcium level adjusted to albumin (mmol/L)	140,278	8.8 ± 0.3	8.8 ± 0.3	< 0.001	9.0 ± 0.3	9.0 ± 0.3	< 0.001
FSH	1380	9.6 ± 14.8	6.8 ± 7.7	< 0.001	64.6 ± 26.3	38.9 ± 34.3	0.0121
Estradiol	2848	156.8 ± 164.5	177.0 ± 192.7	0.019	19.0 ± 55.4	35.8 ± 76.1	0.032
Parous	135,925	86.6	80.0	< 0.001	97.7	93.3	< 0.001
History of estrogen replacement therapy	143,456	1.1	1.1	0.149	4.6	7.0	< 0.001
Breast cancer of family member	143,456	2.9	3.0	0.189	3.8	4.0	0.638
Ovary cancer of family member	143,456	0.5	0.4	0.064	0.8	0.7	0.763
Past history of breast cancer	143,456	0.2	0.1	0.001	1.2	1.2	0.888

Data are presented as mean ± standard deviation or %.

### Association between anthropometric factors, age and SMI

**Figure 1:**
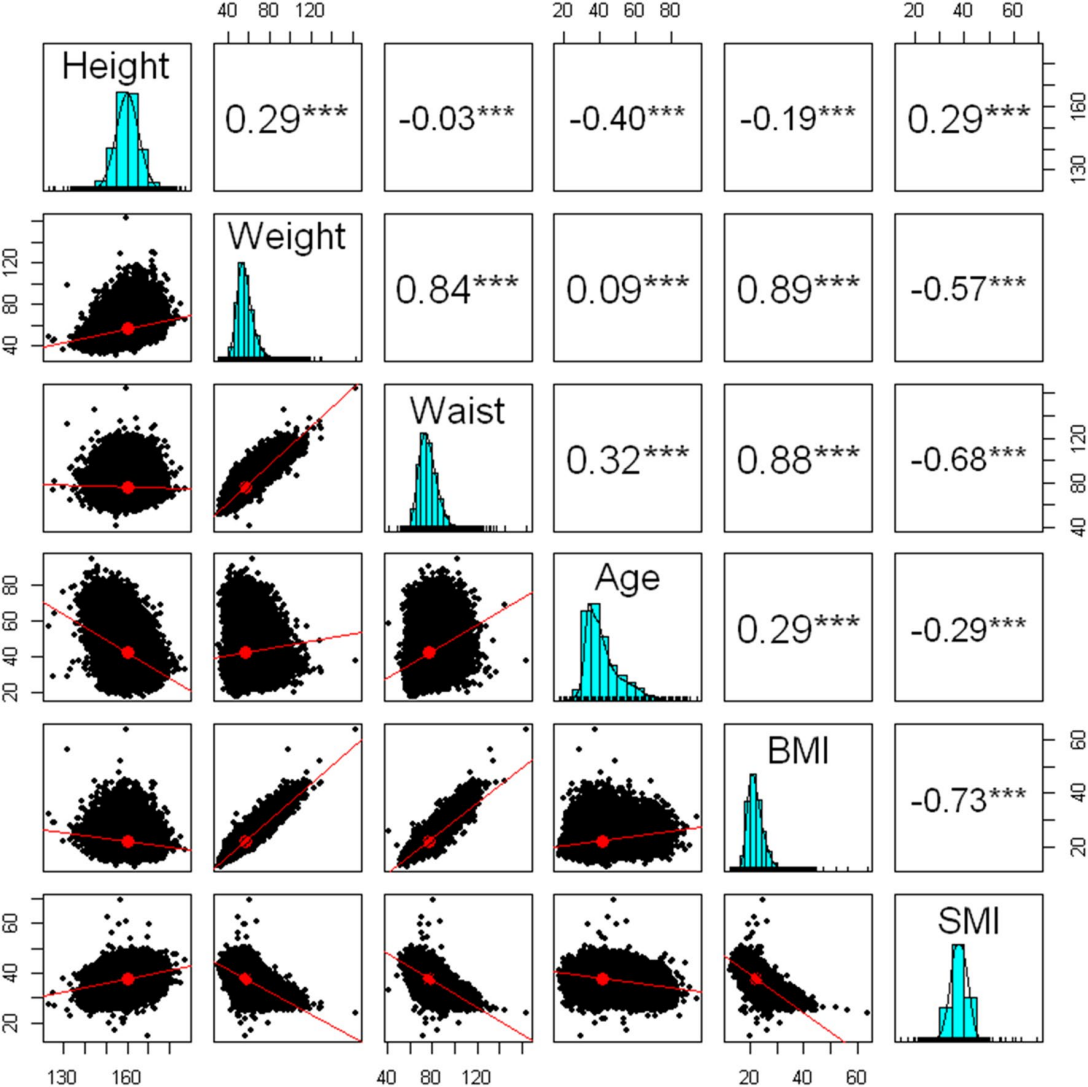
Correlations of anthropometric factors, age and SMI. Scatter plots and Pearson coefficient of Correlation. BMI: body mass index; SMI: skeletal muscle mass index; Waist: Waist circumference.

Pearson correlations and scatter plots between anthropometric factors, age and SMI are presented in Fig. 1. As expected, weight, BMI and waist circumference are strongly related to each other (r = 0.88–0.89), and All of them showed a negative correlation with SMI (r = − 0.73 to − 0.57). In correlation with age, height showed the strongest correlation (r = − 0.4) and weight showed the weakest correlation (r = 0.09), and BMI (r = 0.29), waist circumference (r = 0.32), and SMI (r = − 0.29) showed similar degrees of correlation.

### Logistic regression analysis for known risk factors for dense breasts and anthropometric factors

**Figure 2:**
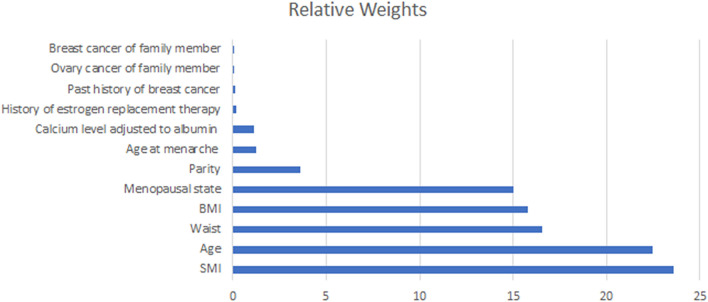
Relative weights for the association of mammographic density with predicting factors. BMI: body mass index; SMI: skeletal muscle mass index; Waist: Waist circumference.

To examine the effect of each factor on MD, we performed a logistic regression analysis of the known risk factors for dense breasts from previous studies and RW analysis. Younger age, nulliparity and low calcium level after adjustment to albumin were significantly associated with dense breasts, regardless menopausal state. Age at menarche, history of estrogen replacement therapy had different effects on dense breasts according to menopausal state (Table 2). RW analysis was used to compare the relative importance of each variable in predicting dense breast (Fig. 2). Among all variables, SMI has the highest RW (RW = 23.62), other anthropometric factors showed relatively high RW values (Waist circumference = 16.55; BMI = 15.78). Age and menopausal state also showed high RW values of 22.74 and 15.02, respectively. Other factors that were known to be related to density had relatively low RW values (Parity = 3.60; Age at menarche = 1.26; Calcium level adjusted to albumin = 1.14; History of estrogen replacement therapy = 0.16; Past history of breast cancer = 0.10; Ovary cancer of family member = 0.02; Breast cancer of family member = 0.01).

**Table 2:** Association between known factors that influence breast density and higher mammographic density by menopausal status.

	Premenopausal women		Postmenopausal women	
	Odds ratio	*p* value	Odds ratio	*p* value
Age (per 1 year increase)	0.94 (0.93–0.94)	< 0.001	0.88 (0.88–0.99)	< 0.001
Age at menarche (14 years)	1.12 (1.10–1.15)	< 0.001	0.58 (0.53–0.64)	< 0.001
Parity	0.62 (0.60–0.64)	< 0.001	0.33 (0.28–0.39)	< 0.001
History of estrogen replacement therapy	0.92 (0.82–1.03)	0.149	1.57 (1.3–1.83)	< 0.001
Calcium level adjusted to albumin (mmol/L) (8.86)	0.92 (0.90–0.94)	0.005	0.74 (0.68–0.80)	< 0.001

Table 3 illustrates the associations between dense breast and anthropometric factors according to menopausal status. Increasing age is well known to be associated with decreasing MD. We therefore corrected age in the analysis of anthropometric factors. Weight, BMI, SMI and waist circumference were statistically significant associated with MD, whereas height was not. These associations remained significant after further adjustment for age at menarche, parity, use of estrogen replacement therapy, and calcium level adjusted to albumin. In both pre and postmenopausal women, weight, BMI, and waist circumference were inversely associated with dense breasts, whereas SMI was positively associated with dense breasts.

**Table 3:** Associations between higher mammographic density and anthropometric factors by menopausal status.

Variables	Premenopausal women Age adjusted OR (95% CI)	Multivariate OR^a^ (95% CI)	Postmenopausal women Age adjusted OR (95% CI)	Multivariate OR^a^ (95% CI)
**Height (cm)**				
Q1 (< 156.4)	Ref	Ref	Ref	Ref
Q2 (156.4–160)	1.00 (0.96–1.03)	0.99 (0.96–1.03)	1.13 (1.02–1.25)	1.16 (1.04–1.30)
Q3 (160–163.6)	0.98 (0.94–1.01)	0.97 (0.93–1.00)	1.07 (0.95–1.19)	1.08 (0.96–1.23)
Q4 (> 163.6) Per 1 cm increase	0.96 (0.93–1.00)	0.94 (0.91–0.98) 0.99(0.99–1.00)	1.03 (0.89–1.18)	1.05 (0.90–1.22) 1.01 (1.00–1.01)
**Weight (kg)**				
Q1 (< 50.9)	Ref	Ref	Ref	Ref
Q2 (50.9–55.4)	0.61 (0.59–0.63)	0.62 (0.60–0.64)	0.61 (0.55–0.68)	0.59 (0.52–0.66)
Q3 (55.4–60.8)	0.42 (0.41–0.43)	0.42 (0.41–0.44)	0.44 (0.39–0.49)	0.42 (0.37–0.48)
Q4 (> 60.8) Per 1 kg increase	0.23 (0.22–0.24)	0.23 (0.23–0.24) 0.93 (0.93–0.93)	0.28 (0.25–0.31)	0.26 (0.23–0.30) 0.93 (0.93–0.94)
**Body mass index (kg/m**^**2**^**)**				
Q1 (< 19.93)	Ref	Ref	Ref	Ref
Q2 (19.93–21.6)	0.53 (0.52–0.55)	0.54 (0.52–0.56)	0.58 (0.51–0.65)	0.58 (0.51–0.67)
Q3 (21.6–23.77)	0.36 (0.35–0.37)	0.37 (0.36–0.38)	0.37 (0.33–0.42)	0.35 (0.31–0.40)
Q4 (> 23.77) Per 1 kg/m^2^ increase	0.19 (0.19–0.20)	0.20 (0.19–0.21) 0.80 (0.80–0.81)	0.23 (0.20–0.26)	0.21 (0.19–0.25) 0.81 (0.80–0.83)
**Skeletal mass index**				
Q1 (< 35.5)	Ref	Ref	Ref	Ref
Q2 (35.5–37.8)	1.80 (1.73–1.87)	1.81 (1.74–1.89)	1.61 (1.44–1.81)	1.59 (1.40–1.82)
Q3 (37.8–40.1)	2.80 (2.70–2.91)	2.80 (2.70–2.91)	2.42 (2.15–2.72)	2.54 (2.23–2.90)
Q4 (> 40.1) Per 1 unit increase	5.37 (5.17–5.58)	5.38 (5.17–5.59) 1.23 (1.23–1.24)	4.51 (4.00–5.08)	4.70 (4.11–5.37) 1.21 (1.19–1.23)
**Waist circumference(cm)**				
Q1 (< 70.5)	Ref	Ref	Ref	Ref
Q2 (70.5–75.5)	0.54 (0.52–0.56)	0.54 (0.53–0.56)	0.50 (0.44–0.57)	0.48 (0.42–0.55)
Q3 (75.5–81.4)	0.36 (0.34–0.37)	0.36 (0.35–0.37)	0.34 (0.30–0.38)	0.32 (0.28–0.36)
Q4 (> 81.4) Per 1 cm increase	0.20 (0.20–0.21)	0.21 (0.20–0.21) 0.92 (0.92–0.93)	0.22 (0.19–0.25)	0.20 (0.18–0.23) 0.93 (0.92–0.93)

^a^Odds ratios (ORs) and 95% confidence interval (95% CI) from the multivariate model including age and all variables (age at menarche, parous, History of estrogen replacement therapy, calcium level adjusted to albumin) presented in the Table 2.

### Associations between breast density and SMI after stratification or adjustment by BMI

To investigate whether SMI was related to MD independent of BMI, we analyzed the association of MD with SMI after further adjustment for BMI. It was seen that SMI was still a statistically significant factor associated with MD. Premenopausal women in the highest quartile of SMI were 2.65 times more likely to have dense breast tissues than those in the lowest quartile (95% CI 2.52–2.79), while postmenopausal women in the highest quartile of SMI were 2.39 times more likely to have dense breast tissues than those in the lowest quartile (95% CI 2.02–2.82) (Table 4).

**Table 4:** Multivariate analysis for the association of higher mammographic density with skeletal mass index after further adjustment for BMI.

	Premenopausal women		Postmenopausal women	
	Multivariate (+ BMI) OR^a^ (95% CI)	*p* value	Multivariate (+ BMI) OR^a^ (95% CI)	*p* value
**Skeletal mass index**				
Q1 (< 35.5)	Ref		Ref	
Q2 (35.5–37.8)	1.26 (1.21–1.32)	< 0.001	1.16 (1.01–1.33)	0.034
Q3 (37.8–40.1)	1.63 (1.55–1.70)	< 0.001	1.55 (1.33–1.80)	< 0.001
Q4 (> 40.1)	2.65 (2.52–2.79)	< 0.001	2.39 (2.02–2.82)	< 0.001
Per 1 unit increase	1.15 (1.15–1.16)	< 0.001	1.13 (1.11–1.15)	< 0.001

^a^Odds ratios (ORs) and 95% confidence interval (95% CI) from the multivariate model including age, body mass index (BMI) and all variables (age at menarche, parous, history of estrogen replacement therapy, calcium level adjusted to albumin) presented in the Table 2.

## Discussion

To our knowledge, this is the first study about the association between skeletal muscularity and MD, and we demonstrated that SMI was the most important predictor among the known predictors of MD by using RW analysis. SMI was also an independent predictor of dense breasts after adjustment for known factors associated with MD, including age and BMI. These results suggest that SMI is a measure to overcome the inconsistency in BMI observed in previous MD-related studies; this could be explained by the strong relationship between skeletal muscularity and breast parenchymal tissue.

In infancy, childhood, puberty, and adulthood, the development of body is affected by a variety of hormones. Among them, gonadal sex steroids, growth hormone and insulin-like growth factor-1 are known to play a major role in development of mammary gland as well as skeletal muscle^25^. Additionally, the role of epidermal growth factor family of growth factors is known in the development of epithelial tissues, particularly the mammary gland and the gastrointestinal tract, and their role in skeletal tissue growth was also reported^26^. Thus, based on this growth mechanism, the relationship between SMI and breast parenchymal tissue is highly relevant, and might explain the relationship with MD.

There are many studies on the relationship between MD and adiposity. Easily measurable but potentially inaccurate estimates of adiposity such as weight, BMI, waist circumference, weight gain have proven to be relevant to MD^5–9^. In our study, as in previous studies, factors representing adiposity, such as BMI, weight, and waist circumference were found to be significantly associated with MD in both pre and postmenopausal women. However, these measurements cannot distinguish between muscle and adipose tissue; hence, it may have a limitation for use as a marker to represent adipose tissue.

Attempts have been made to measure adiposity or muscularity accurately using imaging methods, such as whole-body dual x-ray absorptiometry (DXA) or abdominal computed tomography (CT) scans^6,10,11^. These studies that suggest that body or abdominal fat assessed by DXA or abdominal CT might have an association with the percentage of dense area in breast, independent of BMI. The eight-polar BIA used in our study was reported to be superior to the DXA or body weight used in other studies, for the accuracy of fat mass and muscle mass measurements^27,28^. The SMI measured by BIA indicates the ratio of the skeletal muscle and adipose tissue in the body. The significant association between MD and SMI independent of BMI in our study might further strengthen the results of previous studies, and the SMI measured by BIA could be better indicator of predicting MD.

Based on previous studies, although adiposity seems to be highly related to MD and breast cancer, it is still difficult to understand the biological pathway and has different effect according to menopause state. In early-adulthood, estrogen synthesis in subcutaneous fat accounts for only about 5% of the total, but when the fat mass is excessive, negative feedback is received in the hypothalamic-pituitary-axis^29^. This irregularity of the menstrual cycle induces a decrease in ovarian function, resulting in a decrease of estrogen synthesis. Low level of the sex hormone is associated with inhibition of the growth of the fibroglandular tissue of the breast, resulting in a low MD and a reduced risk of breast cance^19^. However, in late-adulthood, the increase in adiposity due to weight gain increases only the breast fat tissue (non-dense area) without an increase in the fibroglandular tissue (dense area), resulting in a low MD. Although results vary based on the hormones used or the molecular subtype, it is known that obesity in postmenopausal women increases the risk of breast cancer, which is in contrast to the results in premenopausal women^30,31^. It could be because an increase in fat mass caused a higher level of estrogen, induced by aromatization of androstenedione^32^.

Increasing age is strongly associated with declines in MD, which occurs in both pre- and post-menopausal women, regardless of race^15^. Reproductive hormones that decrease with aging affect MD, which is prominent in the premenopausal period^15,33^. In particular, the hormonal changes that occur during the menopausal transition are reflected in the drastic decline in MD^15,34^. In our study, age showed the highest RW value except for SMI, confirming the strong correlation between age and MD. Therefore, in order to more clearly identify the association between SMI and MD, we adjusted for age in analyses.

We also conducted a verification of factors that have been previously associated with MD. Alcohol consumption has been known to be associated with an increased breast cancer and MD in most studies^12,13,35,36^. In our study, postmenopausal women with dense breasts consumed larger amount of alcohol than those with non-dense breasts, while premenopausal women did not. Vitamin D is known to inhibit estrogen synthesis and its receptor function, and inhibit breast epithelial and stromal proliferation, suggesting an inverse relationship with breast cancer and MD^14,37,38^. Based on this theory, several studies have examined the association between MD and vitamin D, but the meta-analysis did not yield significant results^14^. In our study, vitamin D levels were low in women with high MD regardless of the menopausal status. Considering that our study was based on the data from vitamin D measurements of 97,072 participants, which was much larger than the data in a previous meta-analysis (*n* = 5863), this result could be significant. For a clear conclusion, a further study with correction of the confounding factors might be needed.

The limitation of this study is that quantitative measurements were not used for the measurement of MD. Quantitative measurements are expressed as continuous variables, which might provide more accurate statistical results than the classification based on the BI-RADS method that we used. Second, all participants were Koreans, and they have more dense breasts than other races. Therefore, the results cannot be extrapolated to the general population.

We found a positive correlation between the muscularity and breast density. SMI was independent and strong predictor of dense breast. Considering our results and growth mechanism, the proportion of muscularity in the body are likely to be correlated with the proportion of parenchyma in the breast. It offers the possibility to explain the reason for the paradoxical relationship between BMI, MD and breast cancer risk, and for disagreement between previous studies. Further studies are needed to understand the causal link between muscularity, MD and breast cancer risk.

## Data Availability

The datasets generated and analyzed during the current study are available from the corresponding author on reasonable request.

## Abbreviations

BIA: Bioelectrical impedance analyzer
BI-RADS: Breast imaging reporting and data system
BMI: Body mass index
CT: Computed tomography
DXA: Dual x-ray absorptiometry
MD: Mammographic density
OR: Odds ratio
SMI: Skeletal muscle mass index
RW: Relative weight
